# A Case of Occlusion of the Os Uteri

**Published:** 1892-08

**Authors:** William L. Davis

**Affiliations:** Albany, Ga.


### A CASE OF OCCLUSION OF THE OS UTERI.

BY

WILLIAM L. DAVIS, M. D.,

ALBANY, GA.

I was called into the country about fifteen miles to see a patient. While there a negro woman consulted me about herself, wanting to know if she was pregnant. She was about thirty-five years of age, married twice and never had conceived before this time. She was well developed and at that time a picture of health. Prior to this she had suffered with endometritis, leucorrhœa and irregular menstruation. Her old mother (a midwife) told her that she was not pregnant but "conjured" and some enemy of hers had put a snake in her belly. I tried to explain to her that this was impossible, but that she was pregnant beyond the shadow of a doubt. I examined her abdomen and found the fœtal head, also the fœtal heart sound, but still she could not believe she was "in sad health."

About five months from the time I first saw her I was summoned in great haste. When I arrived I found her in labor, made a digital examination, she lying in a dorsal position. Could not find the os uteri. I made several examinations without avail. I then made an ocular examination with a bivalve speculum and failed. Then made her get on the edge of the bed as in instrumental delivery, and examined her while in that position, and also made an examination while standing in an erect position. Still failing, I decided I had a case of atresia oris uteri. I called in my friend, Dr. Bacon. He made the same examination I had with a similar result. We consulted and decided to operate at once. Gave chloroform as an anesthetic. She was placed in a dorsal position, with her hips on the edge of the bed. Passing the index finger high up into the vagina, we found a point where we thought the os should be, and made an incision, dilated and performed craniotomy. Delivered her very quickly and found the placenta had adhered to the walls of the uterus. We were compelled to deliver the placenta by forcible means. The hemorrhage was quite free, but we controlled it readily by the use of ergot hypodermically, and vinegar tampons pushed high up into the vagina. We remained there some time to see the effect. Used carbolic acid as an antiseptic wash. Gave tincture of opium when in pain, oil of ricini as a cathartic the third day. Bowels acted well. Gave chicken essence as a nourishment. I saw her the next day, temperature 99, pulse 85. Her mother was extremely ignorant and a very poor nurse, having given her only one small cup of nourishment during her illness. She lived about five days. Had she resided nearer town and been more easy of access by her physicians, with proper nursing and attention she might have recovered.

Now, what caused the occlusion of the os? I think it was caused by using a rough pointed syringe or some strong irritating injection when she was suffering with leucorrhœa, which irritated the parts, and in healing the adhesion took place. I have asked several physicians of my acquaintance if such a case had ever come under their observation, or if they had read of such an instance. They say not. I have examined several text books and different medical journals, and cannot find a case on record, except in Bedford's work, published by Wm. Wood, edition of 1859. His case was similar to the one described.

